# Flexural Performance Analysis of Composite Beam with Reinforced HPFRCC Precast Shell

**DOI:** 10.3390/ma18040762

**Published:** 2025-02-09

**Authors:** Tingting Lu, Yuxiang Wen, Kai Guan, Bin Wang

**Affiliations:** Shaanxi Key Laboratory of Safety and Durability of Concrete Structures, Xijing University, Xi’an 710123, China; lx549499@163.com (Y.W.); xjdxgkai@163.com (K.G.); b635103208@163.com (B.W.)

**Keywords:** composite beams, R/HPFRCC prefabricated shell, prefabricated monolithic structure, bending performance, bearing capacity calculation model

## Abstract

To enhance the mechanical properties of precast composite beams, High-Performance Fiber Reinforced Cementitious Composite (HPFRCC) material was used instead of ordinary concrete in the precast shell with reinforced bars to form the R/HPFRCC precast shell composite beam. By controlling different reinforcement ratios, post-longitudinal reinforcement treatment methods, mold shell materials, and loading methods, nine test beams were designed, and four-point bending loading tests were carried out to study the flexural bearing capacity, failure mode, failure process, deformation capacity, and influencing factors of composite beams. The R/HPFRCC prefabricated shell composite beams presented good mechanical performance and integrity. Compared with the RC shell composite beams, the R/HPFRCC prefabricated shell composite beam increased the yield and peak loads by 6.6% and 10.3%, respectively. Using HPFRCC material instead of ordinary concrete in the prefabricated shell could reduce the damage degree of the composite beam under bending. Under the same load, the reinforcement strain in the R/HPFRCC precast shell was smaller than that of the RC precast shell and the cast-in-situ RC beam; thus, the yield of longitudinal reinforcement was effectively delayed. Considering the HPFRCC material mechanical properties, a calculated model for the ultimate load-carrying capacity of R/HPFRCC precast shell composite beams was established. The calculated values were in good agreement with the experimental values.

## 1. Introduction

In addition to lowering the gravity load and improving the overall seismic performance of structures, the assembled monolithic composite structure combined the benefits of cast-in-place monolithic and prefabricated structures. It also resolved the hoisting issue of a fully prefabricated structure. Assembled monolithic structures are widely used in reinforcement structures, bridge construction, industrial buildings, residential buildings, and other projects. The enhanced composite components had good bearing capacity and deformation capacity, according to the findings of researchers who worked to increase the mechanical performance of the assembled monolithic composite members [1,2].

The bearing capacity, deformation capacity, and damage resistance of structures could all be enhanced by using High-Performance Fiber Reinforced Cement Composites (HPFRCC) in the anticipated damage site [3]. Some researchers used HPFRCC materials in composite components to improve their integrity and mechanical properties. Engineered Cementitious Composite (ECC)/RC composite beams and their mechanical characteristics were investigated by Liang Jianning’s research group [4]. It showed that applying an ECC layer improved flexural strength and deformability and that the degree of improvement rose as the thickness of the ECC layer grew. Some researchers performed experimental research on the shear strength and deformation capacity of ECC by substituting it for concrete in the compression zone [5,6]. The findings demonstrated that the reinforced concrete (RC) beam’s shear strength and deformation capacity were greatly increased by including ECC in the compression zone. The performance of the flexural synergy impact of RC beams reinforced with HPFRCC was also investigated [7,8]. RC composite beams with the HPFRCC permanent template showed better performance than RC beams in bearing capacity and damage resistance. Several researchers studied the shear property of composite beams with ECC permanent formwork [9,10]. The shear capacity was enhanced by the use of permanent formwork with fewer stirrups. The flexural characteristics of ECC composite beams were investigated by Qin et al. [11]. The beam with the ECC layer exhibited better deformability and energy absorption capability. In terms of material properties, Ma et al. [12] compared the influence of PVA fiber and steel fiber on the flexural properties of cement-based materials. The results showed that PVA fiber could significantly improve the specimen’s deformation capacity under bending load compared with steel fiber. In addition, compared with polyethylene fiber, PVA fiber was more conducive to improving the flexural toughness of the specimen [13,14]. John et al. [15] used the Fabric-reinforced Cementitious Matrix/Mortar (FRCM) system for the reinforcement of RC columns and verified it by FEM simulation and experimental data. The results showed that the FRCM system provided valuable insights for the seismic reinforcement of RC columns. In addition to playing a vital role in structural health assessment and fire-induced damage assessment [16,17], Non-destructive testing (NDT) technology and nondestructive evaluation (NDE) technology could also evaluate the damage evolution and durability of composite beams, especially crack propagation monitoring.

The above studies showed that using HPFRCC as a permanent formwork or for reinforcing reinforced concrete elements to form composite elements could significantly improve the original concrete elements’ bearing capacity and deformation capacity. However, there are few studies on the influence of HPFRCC material on the assembled monolithic structure when it was applied to the prefabricated shell of the assembled structure. In order to improve the mechanical performance of the precast composite components, a new R/HPFRCC prefabricated shell monolithic composite beam was proposed. The prefabricated shell of the monolithic composite beam in this research was made of HPFRCC material. The welded steel mesh and the R/HPFRCC prefabricated shell were poured together. The R/HPFRCC precast shell worked together with the post-cast concrete. Due to the mechanical properties of HPFRCC materials, the flexural properties and bearing capacity calculations of R/HPFRCC prefabricated mold shell composite beams need to be further studied. The flexural failure mode, flexural bearing capacity, failure mechanism, deformation capacity, and influencing variables of R/HPFRCC prefabricated shell monolithic composite beams were investigated through experiments. The flexural bearing capacity of the R/HPFRCC prefabricated shell composite beam was analyzed using a theoretical mechanical method, leading to the establishment of the suggested bearing capacity calculation model. This study will provide a theoretical basis for the engineering application of this structural form.

## 2. Overview of the Experiment

### 2.1. Specimen Design

**Figure 1:**
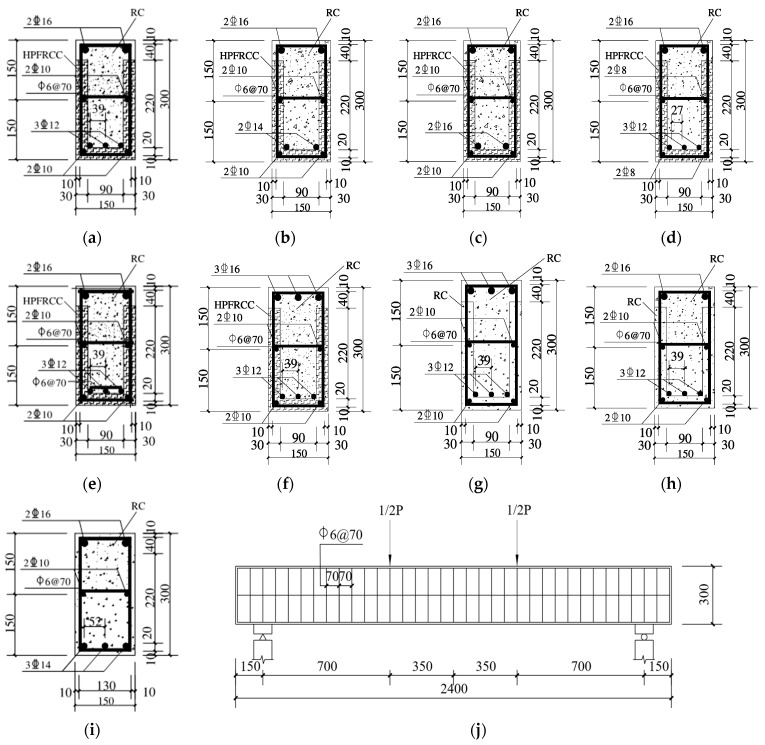
Dimensions of composite beam models. (**a**) HPSTD1.21R; (**b**) HPSTD1.13R; (**c**) HPSTD1.36R; (**d**) HPSTD1.07R; (**e**) HPSTD1.21R-W; (**f**) HPREV1.46R; (**g**) RCREV1.46R; (**h**) RCSTD1.21R; (**i**) ZSTD1.11R; (**j**) Longitudinal section of the beam.

According to the research, the specimen design was based on the 1/2 scale of a prototype structure. In this study, eight composite beam specimens and one integral cast-in-situ RC beam specimen were designed considering the reinforcement ratio, post-longitudinal reinforcement treatment, different mold shell materials, and different loading methods, among which six were prefabricated mold shell composite beams made of HPFRCC material, and their numbers were HPSTD1.21R~HPSTD1.07R, HPSTD1.21R-W, and HPREV1.46R. Among them, HPSTD1.21R-W considered the different treatment methods of longitudinal reinforcement grid in post-cast concrete; HPREV1.46R was a back-loaded specimen; and the other two were RC prefabricated shell composite beams, in which RCSTD1.21R was a comparative analysis specimen with HPSTD1.21R and RCREV1.46R was a comparative analysis specimen with HPREV1.46R. The specimen was 2.4 m in length. According to the research needs, the beam section size was 150 × 300 mm^2^. The thickness of the prefabricated mold shells for HPFRCC and RC was 30 mm. HRB400 and HPB300 grade steel bars, respectively, were used to make the stirrups and longitudinal reinforcements. Table 1 and Figure 1 display R/HPFRCC-constructed monolithic composite beams’ primary design parameters.

**Figure 2:**
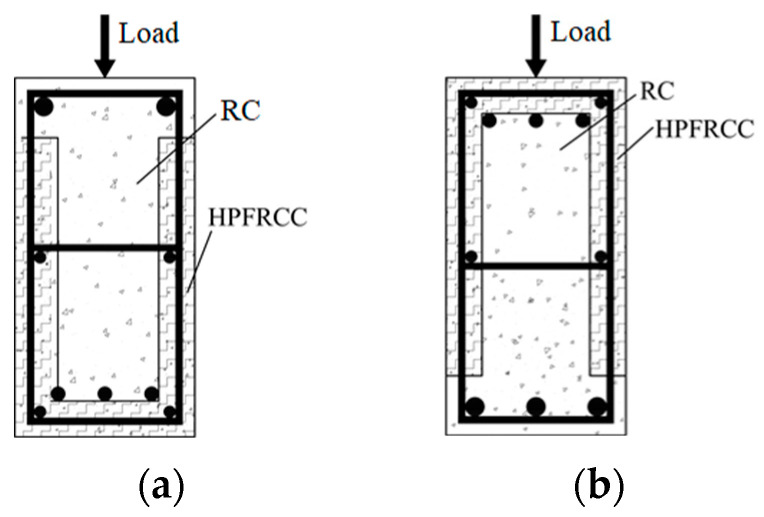
Loading direction. (**a**) The beam bottom was tensioned; (**b**) the beam top was tensioned.

In this test, a reverse load test group was set up, as indicated in Figure 2, since the top section of the beam in the frame construction was susceptible to negative bending moments during earthquakes.

**Figure 3:**
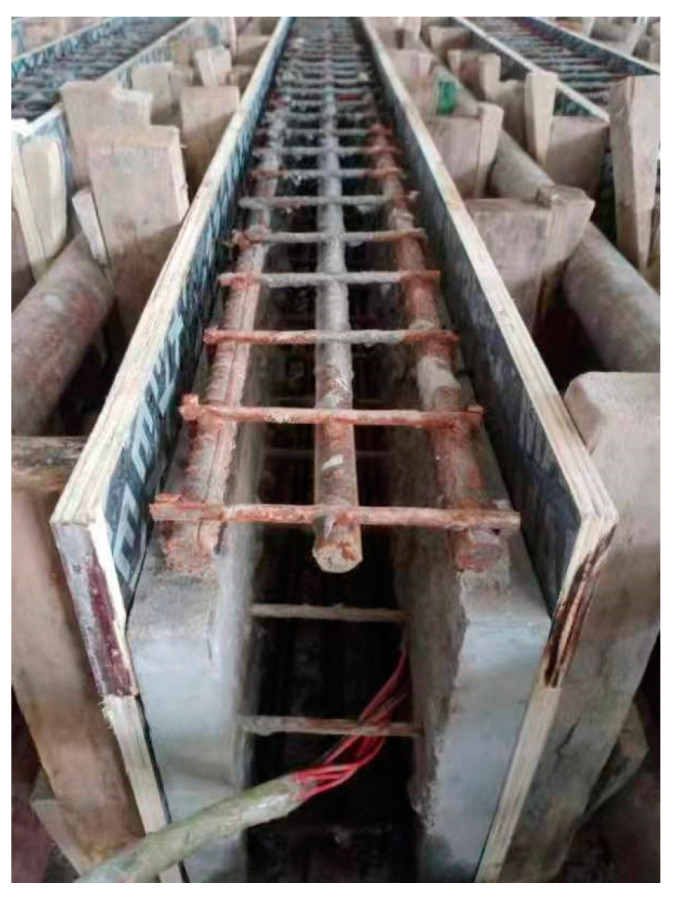
Schematic diagram of the reinforced precast shell.

The pouring process of the specimen is as follows: firstly, the steel mesh was welded and poured together with the U-shaped HPFRCC mold shell or ordinary concrete mold shell. After curing the shell, the conventional concrete was poured with the post-reinforced steel bars, as shown in Figure 3.

### 2.2. Material Properties

**Figure 4:**
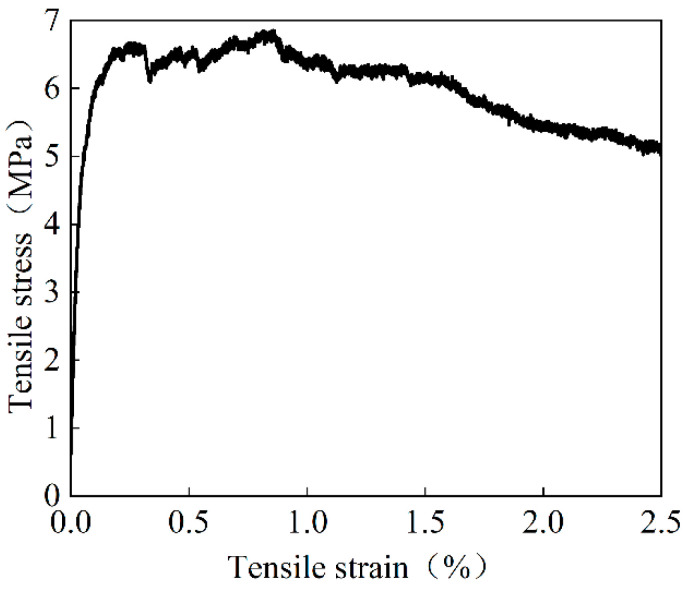
Tensile stress-strain diagram of HPFRCC material [19].

The cement, fly ash, quartz sand (with particles ranging in size from 0.06 to 1.18 mm), PVA fiber, superplasticizer, and water made up the majority of HPFRCC, a cement-based composite material. The HPFRCC material contained 2% of PVA fiber by volume. The polyvinyl alcohol fiber, known as KURALINK-II-12 mm, has the following specifications: 40 μm fiber diameter, 12 mm fiber length, 7% elongation, and 1600 MPa tensile strength. The 100 mm × 100 mm × 100 mm cube blocks and 100 mm × 100 mm × 300 mm prism blocks were tested to obtain a cubic compressive strength of 50.9 MPa and an average axial compressive strength of 40.2 MPa for HPFRCC. The average tensile strength for HPFRCC was 6.85 MPa, as presented in Figure 4. The concrete was made of commercial concrete, and the basic mechanical properties of ordinary concrete were measured according to the standard for test methods of concrete physical and mechanical properties [18]. The average compressive strength of the cube was 49.3 MPa, and the average compressive strength of the prism was 38.2 MPa. The average value of the measured mechanical parameters of the steel bar is shown in Table 2.

### 2.3. Test Setup and Measurement Content

**Figure 5:**
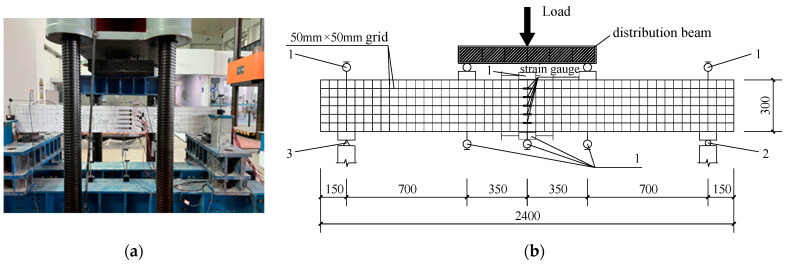
Schematic diagram of the testing device. (**a**) Layout of test setup (beam front); (**b**) Test setup and measurement points (unit: mm, 1—displacement sensor; 2—Hing support; 3—Fixed hinge support).

The vertical load of this test was conducted through a 5000 kN pressure testing machine. Figure 5 is a schematic diagram of the beam flexural performance test device. A 700 mm pure bending part in the span of the beam was formed by the two-point force loading, and the load was distributed by a distribution beam. The pressure sensor on the testing apparatus itself gathered the test load.

The test loading started with load control, with a loading rate of 0.2 kN/s, held at each load level for 300 s until it reached 30 kN. Then, the loading switched to displacement control, with a rate of 0.5 mm/min, held at each displacement level for 600 s; when the displacement reached 30 mm, it switched to 5 mm per level, held for 600 s, until the displacement loading reached 60 mm and stopped.

On the concrete’s side, strain gauges were positioned, and the specimen surfaces’ variations in concrete strain were observed. The change of the neutral axis height and whether it conformed to the plane section assumption were analyzed. The longitudinal reinforcement and the stirrups of the beams were monitored for changes in strain using reinforcement strain gauges. With the displacement meter, the deflection of the composite beams, the cross-section curvature, and the displacement at the support were all measured. The changes in crack width and length were recorded in detail by a high-power crack observation instrument(Haichuang Hi-Tech Technology Co., Ltd, Beijing, CN), and the crack map was taken.

## 3. Results and Analysis

### 3.1. Damage State and Crack Analysis

**Figure 6:**
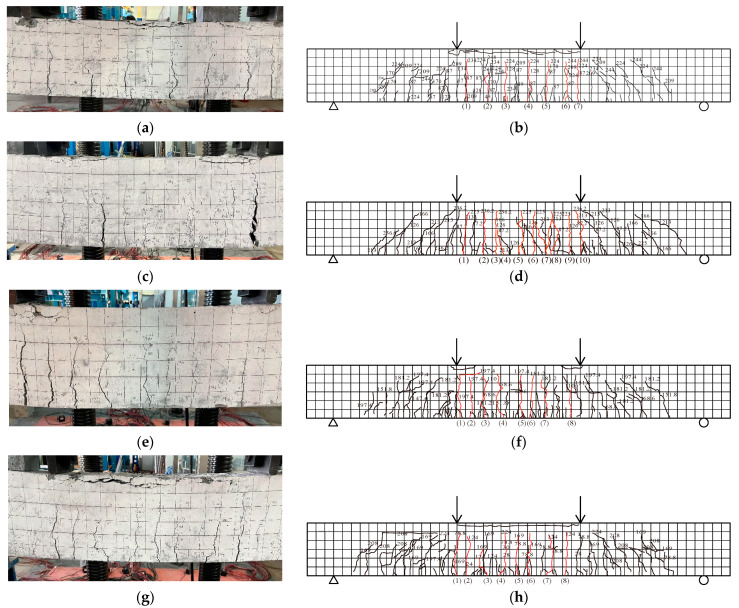

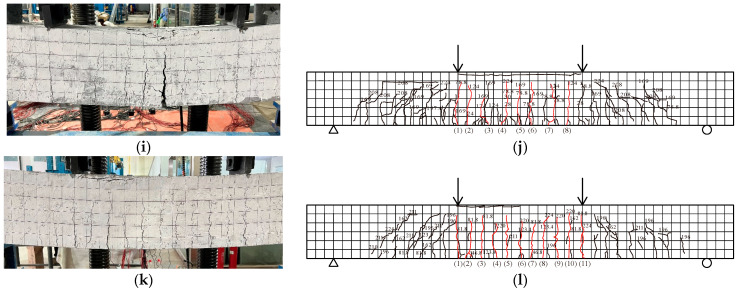
Fracture state and crack distribution. (**a**) HPSTD1.21R failure state; (**b**) HPSTD1.21R crack distribution; (**c**) RCSTD1.21R failure state; (**d**) RCSTD1.21R crack distribution; (**e**) HPSTD1.13R failure state; (**f**) HPSTD1.13R crack distribution; (**g**) HPSTD1.36R failure state; (**h**) HPSTD1.36R crack distribution; (**i**) HPSTD1.07R failure state; (**j**) HPSTD1.07R crack distribution; (**k**) HPSTD1.21R-W failure state; (**l**) HPSTD1.21R-W crack distribution.

Specimen HPSTD1.21R developed cracks for the first time when it was loaded to 18.0 kN, and, as the loading rose, the microcracks in the beam progressively increased. The cracks began to extend upwards at a load of 87.0 kN. At this point, the cracks did not have noticeable width. Then, the beam reached the yielding stage at 219.2 kN of load.The crack had a noticeable width of 0.2 mm at a load of 224.0kN. At 256.6 kN of load, the experiment was stopped due to the large deformation, and the highest crack width in the beam’s frontal side span was 4.1 mm (Figure 6a). Figure 6b illustrates the distribution of the cracks in the beam. When specimen beam RCSTD1.21R was loaded to 28.2 kN, cracks initially appeared with the max length of 70.12 mm. With the increase of loading, although new cracks appeared, they rapidly extended upward in the length of the original cracks. The crack had a noticeable width of 0.21 mm at a load of 126.0 kN, and the cracks extended upward quickly. When loaded to 205.6 kN, the specimen entered the yielding stage. The max load of RCSTD1.21R reached 232.7 kN. When the test stopped, the maximum crack width reached 14.0 mm (Figure 6c). The crack distribution is shown in Figure 6d. Specimen HPSTD1.21R exhibited finer and more cracks in R/HPFRCC assembled monolithic composite beams compared with specimen RCSTD1.21R when the load reached the yield load. It was shown that HPFRCC had excellent crack width control ability and enhanced damage resistance of the RC precast beam. Also, the above-mentioned works of literature [8,15] used UHPC as a permanent formwork or reinforced material on concrete beams to improve the bearing capacity of the components, but the detachment and spalling of the formwork and the reinforcement part were obvious. The specimens proposed in this study showed good overall performance; there was no obvious slippage between the prefabricated shell and the post-cast concrete; and the damage resistance and deformation ability were significantly improved.

When specimen HPSTD1.13R was loaded up to 18.0 kN, cracks first occurred in the tensile zone. Also, as the load increased, the microcracks gradually appeared. At 174.5 kN, the beam entered the yielding stage. Then, the crack developed a noticeable width of 0.2 mm at a load of 197.4 kN. The max load of specimen HPSTD1.13R reached 226.0 kN. When the test stopped, the crack width was 8.3 mm (Figure 6e). Figure 6f illustrates the distribution of the cracks. When specimen beam HPSTD1.36R was loaded up to 12.0 kN, cracks appeared. As the loading rose, the number of microcracks in the beam progressively increased. When loaded to 225.2 kN, the specimen entered the yielding stage. The crack began to develop with a measurable width of 0.2 mm at 229.8 kN. The measured max load of specimen HPSTD1.36R was 267.6 kN. The crack width was 3.8 mm when the test stopped (Figure 6g). Its failure state is displayed in Figure 6h. Specimen HPSTD1.07R first developed cracks in the tensile region when it was subjected to 16.0 kN. The crack development of the specimen was relatively fast. The specimen reached the yield stage at 178.5 kN. The crack width was 0.2 mm at 192.0 kN. The specimen reached its peak load at 219.4 kN with a crack width of 3.1 mm. When the test stopped, the maximum width reached 18.4 mm (Figure 6i). Figure 6j shows the spread of the cracks. As the longitudinal reinforcement ratio *ρ*_l_ grew, so did the R/HPFRCC composite beams’ yield load and ultimate load. It showed that, under the same load, the crack width of the R/HPFRCC composite beam decreased with increasing longitudinal reinforcement ratio.

As can be seen in Figure 6f, the cracks initially developed for specimen HPSTD1.21R-W at 17.6 kN. The specimen went into yielding with a load of 208.3 kN. The largest crack width measured at max load 249.4 kN was 4.0 mm (Figure 6k). It showed that the addition of a transverse reinforcement connection on the post-positioned longitudinal reinforcement had no obvious effect on the yield load or the maximum load, and the control ability of crack development was also similar to that of specimen HPSTD1.21R.

### 3.2. Load-Mid-Span Deflection Curve

**Figure 7:**
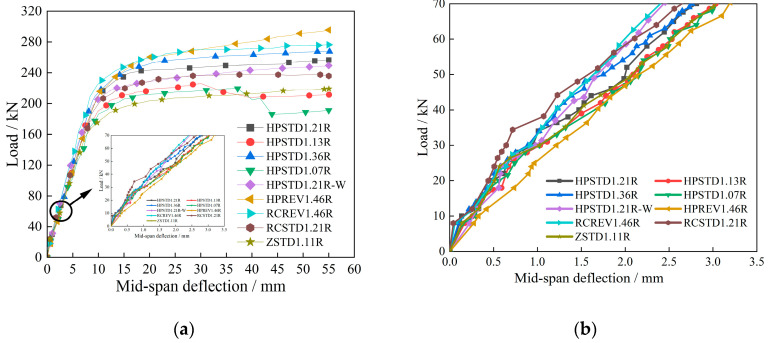
Load-deflection curves of specimens. (**a**) Load-deflection curves of specimens; (**b**) enlarged segment curves.

The displacement sensor was placed to monitor the deflection, and the testing machine was used to gather the load value. Figure 7 displays the load-deflection curves that were acquired during the test. Figure 7 clearly shows the load-deflection curves of the beams.

The first stage was the elastic stage before cracking, during which the stiffness of the specimen beam was at its largest, and the slope of the curve was also at its largest. Every test beam’s mid-span deflection varied linearly as the load increased. Following the emergence of the fracture, the specimen beam’s stiffness reduced, and it moved into the elastoplastic stage. Additionally, exhibiting a nonlinear alteration, the curve’s slope steadily dropped. Because of the strain-hardening property of the HPFRCC materials and the fact that the materials still bore some tensile tension at the time the crack initially emerged, the beam curve with the R/HPFRCC prefabricated shell would not have a visible turning point after breaking. The test beam developed more cracks as the load was gradually raised, and the crack breadth also kept growing. Because the elastic modulus of HPFRCC was lower than that of conventional concrete during the elastic stage, the load-deflection curve of specimen RCSTD1.21R had a slightly greater slope than that of specimen HPSTD1.21R. It indicated that the initial stiffness of the beam RCSTD1.21R was greater than that of the beam HPSTD1.21R. For R/HPFRCC composite beams (HPSTD1.21R, HPSTD1.13R, HPSTD1.36R, and HPSTD1.07R), the mid-span deflection curves were nearly identical, and for R/HPFRCC composite beams (HPSTD1.21R-W) with reinforcement mesh, they were nearly identical to those of HPFRCC composite beams (HPSTD1.21R) with rear longitudinal reinforcement.

In the second stage, the R/HPFRCC composite beams (HPSTD1.21R, HPSTD1.13R, HPSTD1.36R, and HPSTD1.07R) with different reinforcement ratios exhibited a progressive rise in slope. Furthermore, because of its post-positioned longitudinal reinforced bars mesh, the R/HPFRCC composite beam (HPSTD1.21R-W) had a larger curve slope than the R/HPFRCC composite beam (HPSTD1.21R). When under reverse loading, the ordinary RC composite beam (RCREV1.46R) experienced a steeper and larger load-mid-span deflection curve due to its higher concrete stiffness compared to HPFRCC. Consequently, the RCREV1.46R underwent less deformation under the same load than the HPREV1.46R. Before the reinforcement yielded, the beam’s (HPSTD1.21R) deflection curve progressively increased in slope compared to that of specimen RCSTD1.21R and specimen ZSTD1.11R. It indicated that the HPFRCC could cooperate with the reinforcement to bear the tensile force together. This could potentially postpone the tensile longitudinal reinforcement’s yield and increase the test beam’s yield load.

The test beam progressively moved into the plastic stage after the steel bar yielded. The deflection increased quickly after yielding the large deformation stage, and the load-span deflection curve’s slope gradually decreased and tended to be horizontal. The test beam’s stiffness also gradually decreased, and the R/HPFRCC composite beam prevented the material from cracking for the action of fibers. Specimen HPSTD1.21R had a steeper load-deflection curve than specimens RCSTD1.21R and ZSTD1.11R.

The remaining specimen beams’ bearing capacities did not appreciably rise during the failure stage, except for the two that were reversely loaded (HPREV1.46R and RCREV1.46R). However, during that stage, the neutralization axis shifted upward, the displacement grew, and the length and width of the cracks in each specimen beam increased. The HPFRCC was placed in the compression region after the specimen beam’s capacity to support the ultimate bearing capacity was attained, which helped to increase the structure’s flexural bearing capacity.

As shown in Figure 7, the bearing capacity of specimen HPSTD1.21R employing an R/HPFRCC prefabricated shell was much greater than that of specimen ZSTD1.11R and specimen RCSTD1.21R. Furthermore, the HPFRCC composite beam’s (HPSTD1.21R) mid-span displacement under the class load was smaller than that of specimens RCSTD1.21R and ZSTD1.11R.

Table 3 shows the yield deflection and the measured characteristic load values for each of the nine test beams. Compared to specimens RCSTD1.21R and ZSTD1.11R, the composite beams using R/HPFRCC prefabricated shells had a substantially greater yield load and bearing capacity.

The yield load and peak load of specimen HPSTD1.21R were 6.6% and 10.3% higher, respectively, than the ordinary RC composite beam (RCSTD1.21R) when different pouring techniques were used. Similarly, the peak load and yield load of beam HPSTD1.21R were 24.5% and 17.2% higher, respectively, than the ordinary overall cast-in-situ (ZSTD1.11R).

The yield load and peak load of specimen HPSTD1.13R were found to be 25.6% and 13.5% less than those of beam HPSTD1.21R, and the factor *ρ*_l_ of specimen HPSTD1.21R was 0.08% higher than specimen HPSTD1.13R. The ratio *ρ*_l_ of specimen HPSTD1.36R increased by 0.15%. It also had a yield load and peak load that were 6.5% and 13.5% higher than beam HPSTD1.21R, respectively. Beam HPSTD1.21R’s yield load and peak load were 22.8% and 17.0% greater, respectively, than those of the R/HPFRCC composite beam (HPSTD1.07R), and its longitudinal reinforcement ratio was 0.14% higher.

For the R/HPFRCC composite beam (HPSTD1.21R-W), which used reinforcement mesh as the longitudinal reinforcement of the cast-in-situ portion of the composite beam, the loading system was not identified as the trial stage because it was the first test, and the displacement loading was stopped after loading only 50 mm. Additionally, because the beam HPSTD1.21R-W was made with a construction error at first, the beam had to be reproduced. However, beam HPSTD1.21R-W ’s yield load and peak load were 5.2% and 2.89%, respectively, lower than those of beam HPSTD1.21R. It may be irrational to compare them to those of other beams. The test’s data may be accumulated as test data only.

When the same reverse load was applied, the yield load and peak load of the R/HPFRCC specimen HPREV1.46R were 10.51% and 6.95% higher than those of the regular RC composite beam (RCREV1.46R). It suggested that the placement of HPFRCC in the compression area could, to some extent, increase the structure’s flexural bearing capacity.

A thorough investigation revealed that employing HPFRCC material may increase the structure’s flexural bearing capacity. Increasing the factor *ρ*_l_ could also increase the structure’s bearing capacity.

### 3.3. Moment Curvature Curve Analysis

**Figure 8:**
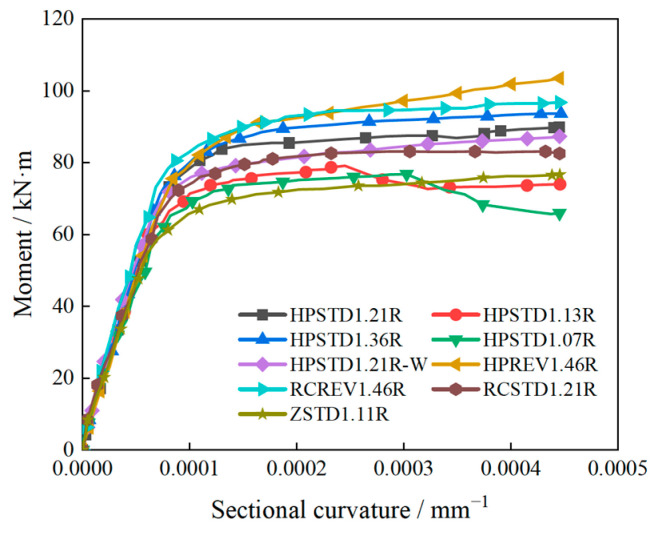
Moment-curvature of section.

The specimen’s bending moment-section curvature relationship is displayed in Figure 8. All the test beams underwent three stages of bending: uncracked, cracked work, and failure. The link between the bending moment and the curvature of the cross-section at various points during the bending process was demonstrated by the R/HPFRCC assembled monolithic composite beam.

The R/HPFRCC assembled monolithic composite beam performed well throughout the uncracked stage when the bending moment and the cross-section curvature displayed a basic linear relationship and no visible cracks were present throughout the bending member. Both the cross-section curvature and the cross-section deflection climbed fast during the cracking stage, and there was a nonlinear connection between the moment and the cross-section curvature. Nevertheless, because of the properties of the HPFRCC material, the fiber material kept functioning even after the fracture appeared, assisting the steel bar in bearing the tensile strain. This successfully increased the component’s resistance to damage. Finally, the flexural component’s failure phase started. The bending moment-section curvature relationship gradually tended to be horizontal, indicating that the flexural member was approaching the failure state. The longitudinally tensile steel bar yields first, followed by the instantaneous breaking of the concrete under high pressure in the compression area, which causes the component to fail plastically quickly. Before the tensile reinforcement yields, the bending moment-section curvature curves were close to different longitudinal reinforcement ratios, different prefabricated mold shell materials, cast-in-situ or not, and other factors. The concrete at the tension position stopped being useful at the crack during the second stage of crack growth. Still, the longitudinal tensile steel bar and fiber material jointly bear the tensile force. The bending moment had a curve relationship with the curvature of the section, and the curvature and deflection of the section increased rapidly. A horizontal straight line appears in the bending moment-curvature relationship when the longitudinally tensile reinforcement gives way in the third failure stage. This was because the concrete in compression was rapidly exposed to high pressure, which led to the failure of the cross-section.

The curvature of the R/HPFRCC composite beam (HPSTD1.21R), normal RC composite beam (RCSTD1.21R), and regular monolithic cast-in-situ RC beam (ZSTD1.11R) was compared. The results showed that specimen HPSTD1.21R performed better in terms of damage resistance because its curvature was smaller under the same bending moment. This could be because of the properties of the HPFRCC material in specimen HPSTD1.21R. The HPFRCC and the longitudinal tensile steel bar cooperated to sustain the tensile force that occurred following the crack, thereby delaying the longitudinal steel bar’s yield and enhancing the component’s resistance to damage.

The R/HPFRCC composite beam (HPSTD1.21R-W) for the rear longitudinal reinforcement had a curvature that was less than beam HPSTD1.21R, suggesting that the longitudinal reinforcement might somewhat increase the structure’s resistance to damage.

By comparing the R/HPFRCC composite beams with various longitudinal reinforcement ratios (HPSTD1.21R, HPSTD1.13R, HPSTD1.36R, and HPSTD1.07R), it was possible to determine that specimen HPSTD1.36R performed better in terms of damage resistance because its curvature was smaller. This revealed that the components’ resistance to damage may be enhanced in some situations by raising the factor *ρ*_l_.

In the event of reverse loading, the curvature of specimen HPREV1.46R was greater than that of specimen RCREV1.46R. It was discovered that the HPFRCC arrangement in the tension zone improved the structure’s damage resistance and that the HPFRCC configuration in the compression zone enhanced the flexural bearing capacity of the structure.

In conclusion, the R/HPFRCC assembled monolithic composite beam’s bending moment-section curvature curve revealed clear phased features, and its special material qualities allowed it to present better performance throughout the crack stage.

### 3.4. Plane Section Assumption

**Figure 9:**
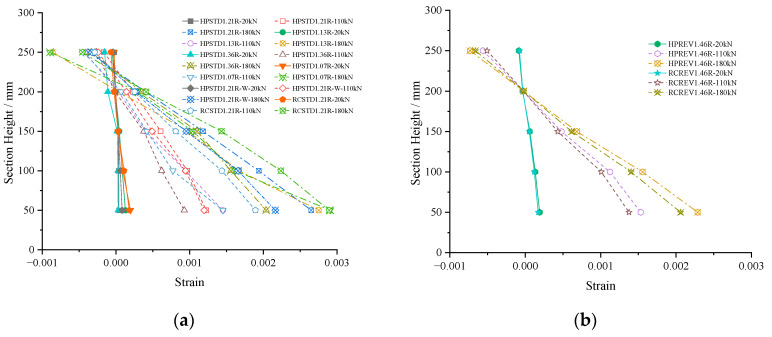
Strain curves along the beam height. (**a**) Composite beams with bottom in tension; (**b**) composite beam with top in tension.

The mechanical analysis of the R/HPFRCC precast shell composite beam flexural bearing capacity is based on the plane section assumption theory. In this experiment, five concrete strain gauges (see Figure 5b) were arranged at equal distances in the middle section of the composite beams to monitor the strain change of the shell material. The measured strain gauge data were plotted along the beam height, as shown in Figure 9a. The strain values of specimen HPSTD1.21R (the shell was HPFRCC material) were slightly smaller than that of specimen RCSTD1.21R. When the beam top was under tension, the strain of the specimen HPREV1.46R was also a little smaller than that of RCREV1.46R (Figure 9b). As the load increased, cracks continued to appear, and the measured strain values were partially deviated and no longer in a completely linear state. However, from an overall trend perspective, the strain values varied approximately linearly along the beam height. This was in line with the plant section assumption and provided a basis for the theoretical and mechanical analysis of the subsequent flexural bearing capacity of the R/HPFRCC composite beams.

## 4. Load-Bearing Capacity Calculation Model

### 4.1. Assumption

It was discovered that the mechanical failure process of R/HPFRCC prefabricated shell monolithic composite beams was comparable to that of integrally poured RC beams through the tests. From the start of loading to the test beam failure, the process was composed of three stages: the uncracked stage, the cracking stage, and the failure stage. The following assumptions were established to analyze the flexural capacity of the cross-section of the HPFRCC precast monolithic composite beam.

The sectional strain remained in the same plane after the member was stressed. The test demonstrated that the concrete strain at each measurement site along the test beam’s cross-section height direction was maintained in the same plane before the test beam’s first fracture appeared, satisfying the plane section assumption. It was concluded that the longitudinal strain of the test beam varied linearly at different load levels based on the data collected by the concrete strain gauges at the time of the crack’s appearance, suggesting that the flat cross-section assumption held. Therefore, the flat section assumption was still applicable for HPFRCC monolithic composite beams.

Load-bearing capacity calculations focus on the strength of the structure under ultimate limits and state that, while creep and shrinkage primarily affect long-term deformation and stress redistribution, they have minimal impact on ultimate-load-bearing capacity, and load-bearing capacity calculations address short-term load responses. For neglecting creep and shrinkage to simplify the calculation process, it had not been taken into account to what extent secondary effects, including the shrinkage and creep of HPFRCC and concrete, affect the internal forces of the members.

Relative slip between HPFRCC and concrete, and between HPFRCC, concrete, and reinforcement, was not considered.

The tensile strength of the concrete after cracking was ignored; that is, concrete in the tensile zone loses its tensile ability soon after cracking. After cracking, the strain-hardening properties of HPFRCC were taken into account, allowing it to sustain the load.

### 4.2. Material Constitutive Relationship

#### 4.2.1. Tensile-Compressive Principal Modeling of HPFRCC

**Figure 10:**
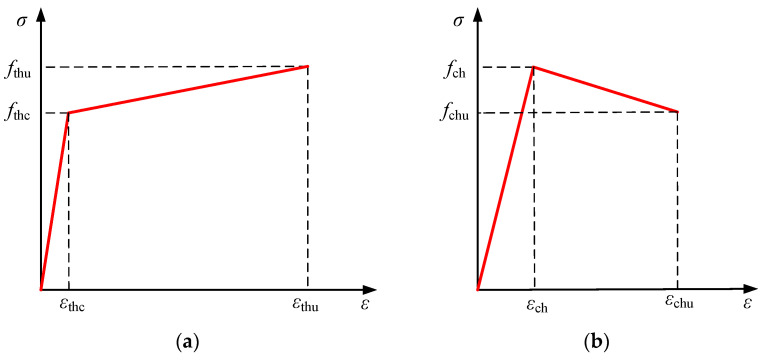
Constitutive modeling of HPFRCC materials. (**a**) Uniaxial tensile curve; (**b**) uniaxial compression curve.

As demonstrated in Figure 10, the tensile and compressive constitutive relation of HPFRCC materials with tensile strain hardening capabilities could be determined using the tensile and compressive constitutive relation given in the literature [20].

The uniaxial tensile stress-strain relationship for HPFRCC was as follows:

(1) $$\sigma_{\mathrm{th}}=\left\{\begin{array}{c} \frac{f_{\mathrm{thc}}}{\varepsilon_{\mathrm{thc}}}\varepsilon_{t}\;0\leq \varepsilon_{t}\leq \varepsilon_{\mathrm{thc}}\\ f_{\mathrm{thc}}+\frac{f_{\mathrm{thu}}-f_{\mathrm{thc}}}{\varepsilon_{\mathrm{thu}}-\varepsilon_{\mathrm{thc}}}\left(\varepsilon_{t}-\varepsilon_{\mathrm{thc}}\right)\;\varepsilon_{\mathrm{thc}}\text{<}\varepsilon_{t}\leq \varepsilon_{\mathrm{thu}} \end{array}\right.$$

where:

*f*_thc_—HPFRCC tensile initial cracking strength;

*ε*_thc_—HPFRCC tensile initial strain;

*f*_thu_—HPFRCC ultimate tensile strength;

*ε*_thu_—HPFRCC ultimate tensile strain.

The uniaxial compressive stress-strain relationship for HPFRCC was as follows:

(2) $$\sigma_{\mathrm{ch}}=\left\{\begin{array}{c} \frac{f_{\mathrm{ch}}}{\varepsilon_{\mathrm{ch}}}\varepsilon_{c}\;0\leq \varepsilon_{c}\leq \varepsilon_{\mathrm{ch}}\\ f_{\mathrm{ch}}+\frac{f_{\mathrm{chu}}-f_{\mathrm{ch}}}{\varepsilon_{\mathrm{chu}}-\varepsilon_{\mathrm{ch}}}\left(\varepsilon_{c}-\varepsilon_{\mathrm{ch}}\right)\;\varepsilon_{\mathrm{ch}}\text{<}\varepsilon_{c}\leq \varepsilon_{\mathrm{chu}} \end{array}\right.$$

where:

*f*_ch_—HPFRCC strength;

*ε*_ch_—HPFRCC strain corresponding to *f*_ch_;

*f*_chu_—HPFRCC ultimate compressive stress value;

*ε*_chu_—HPFRCC strain corresponding to *f*_chu_.

#### 4.2.2. Tensile-Compressive Principal Modeling of Concrete

**Figure 11:**
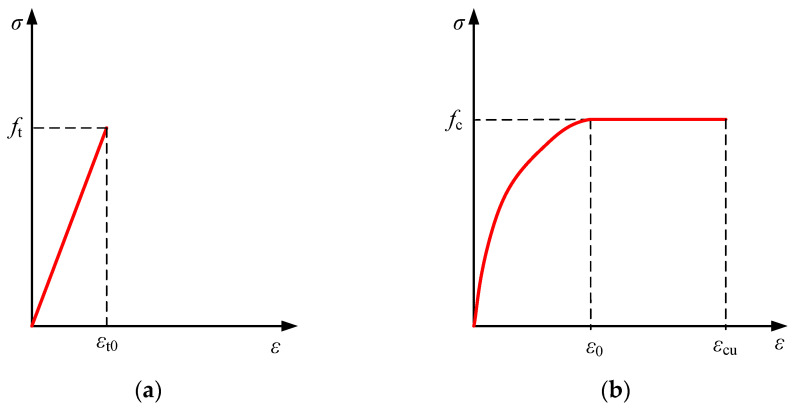
Stress-strain curve of concrete. (**a**) Uniaxial tensile curve; (**b**) uniaxial compression curve.

The Senz model, the German Rüsch model, and the E. Hongnestad model were all often employed. Several researchers [21,22] also established the uniaxial compressive stress-strain model of concrete confined by transverse steel bars and studied the influence of stirrups on the stress-strain relationship of concrete. According to the needs of the research in this paper, the Rüsch model was chosen for mechanical analysis by combining the best features of each model; Figure 11 illustrates the stress-strain model of concrete.

Assuming that concrete in uniaxial tension rapidly lost its tensile capacity after cracking and was immediately withdrawn from work, the stress-strain model for concrete in tension was as follows:

(3) $$\sigma_{\mathrm{ct}}=\left\{\begin{array}{l} \frac{f_{t}}{\varepsilon_{t0}}\varepsilon_{t}\;\;0\leq \varepsilon_{t}\leq \varepsilon_{t0}\\ 0\;\varepsilon_{t0}<\varepsilon_{t} \end{array}\right.$$

where:

*f*_t_—concrete cracking stress;

*ε*_t0_—concrete cracking strain;

The uniaxial compressive stress-strain relationship for concrete was as follows:

(4) $$\sigma_{\mathrm{cc}}=\left\{\begin{array}{ll} f_{c}\left[\frac{2\varepsilon_{c}}{\varepsilon_{0}}-{\left(\frac{\varepsilon_{c}}{\varepsilon_{0}}\right)}^{2}\right] & 0\leq \varepsilon_{c}\leq \varepsilon_{0}\\ f_{c} & \varepsilon_{0}<\varepsilon_{c}\leq \varepsilon_{\mathrm{cu}} \end{array}\right.$$

where:

*f*_c_—compressive strength of concrete;

*ε*_0_—compressive strain of concrete corresponding to *f*_c_;

*ε*_cu_—the ultimate compressive strain of concrete;

#### 4.2.3. Tension-Compression Intrinsic Modeling of Reinforcing Bars

**Figure 12:**
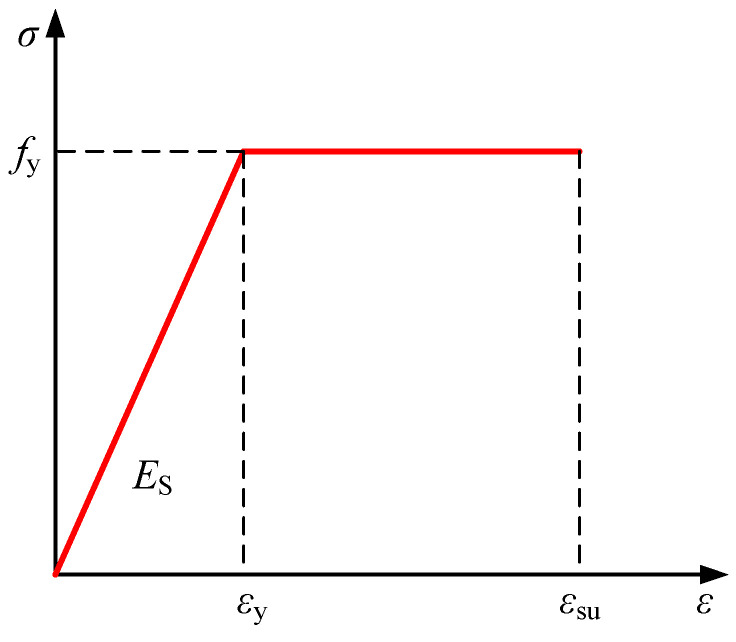
Stress-strain curve of reinforcement.

In this article, the ideal elastic-plastic bilinear model was utilized to analyze the mechanical properties of the steel bars. The intrinsic relationship curve is shown in Figure 12.

The following is the formulation for the reinforcement’s stress-strain relationship:

(5) $$\sigma_{s}=\left\{\begin{array}{ll} E_{s}\varepsilon_{s}, & 0\leq \varepsilon_{s}\leq \varepsilon_{y}\\ f_{y}, & \varepsilon_{y}\leq \varepsilon_{s}\leq \varepsilon_{\mathrm{su}} \end{array}\right.$$

where:

*E*_s_—modulus of elasticity of reinforcement;

*f*_y_—yield strength of reinforcing bars;

*ε*_y_—yield strain of reinforcing bars, *ε*_y_ = *f*_y_/*E*_s_;

*ε*_su_—an ultimate tensile strain of reinforcement;

### 4.3. Calculation of Ultimate Bearing Capacity

When the R/HPFRCC assembled monolithic composite beam was damaged, the HPFRCC in the tensile zone could still bear tensile stress after cracking due to the action of the strain hardening properties. Meanwhile, the compressive concrete reached the maximum compressive strain *ε*_cu_, and the maximum compressive stress of *f*_c_. The maximum tensile strain *ε*_t_ of HPFRCC was between *ε*_thc_ and *ε*_thu_, and the maximum tensile stress *σ*_ht_ was between *f*_thc_ and *f*_thu_. The sign *f*_thc_ was used as the tensile strength of HPFRCC.

Since the height of the mold shell was 250 mm and the beam height was 300 mm, the neutral axis needed to be considered in two cases.

- Neutralization shaft in R/HPFRCC precast shell (height of pressurized zone *x*_c_ ≥ 50 mm)

**Figure 13:**
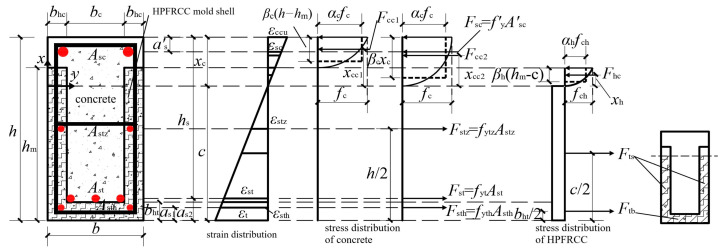
Calculation diagram of bending bearing capacity of R/HPFRCC composite beam under normal section (*x*_c_ ≥ 50 mm).

From the basic assumptions in Section 4.1, for R/HPFRCC precast monolithic composite beams, considering the role of HPFRCC in the tensile zone, the destruction of the cross-section occurred on the premise that the concrete at the edges of the compression zone reached the ultimate compressive strain. Figure 13 represents the stress state and strain of the cross-section at its destruction, from which the ultimate capacity in bending, *M*_u_, could be derived.

According to the force equilibrium condition in Figure 13, the following expression could be obtained:

(6) $$F_{\mathrm{cc}}+F_{\mathrm{hc}}+F_{\mathrm{sc}}=F_{\mathrm{ts}}+F_{\mathrm{tb}}+F_{\mathrm{stz}}+F_{\mathrm{st}}+F_{\mathrm{sth}}$$

(7) $$F_{\mathrm{cc}}+F_{\mathrm{hc}}+f_{y}^{\prime }A_{\mathrm{sc}}^{\prime }=F_{\mathrm{ts}}+F_{\mathrm{tb}}+f_{\mathrm{ytz}}A_{\mathrm{stz}}+f_{\mathrm{yt}}A_{\mathrm{st}}+f_{\mathrm{yth}}A_{\mathrm{sth}}$$

where *F*_cc_ was the concrete stress in the compression zone; *F*_hc_ was the HPFRCC stress in the pressure zone; *F*_sc_ was the tensile force of reinforcement in the compression zone; *f’*_y_ and *A’*_sc_ were the compressive yield stress of the reinforced bars and the area of the reinforced bars, respectively; *F*_stz_ was the tensile force of reinforcement in the middle of the tension zone; *f*_ytz_ and *A*_stz_ were the yield stress of the reinforcement in the middle of the tension zone and the area of the reinforcement, respectively; *F*_st_ was the tension of the post-positioned longitudinal reinforced bars; *f*_yt_ and *A*_st_ were the yield stress of the post-positioned bars in the tension zone and the area of the bars, respectively; *F*_sth_ was the tensile force of reinforcement in the R/HPFRCC mold shell in the tensile zone; *f*_yth_ and *A*_sth_ were the yield stress of the reinforcement in the R/HPFRCC mold shell in the tension zone and the total area of the reinforcement, respectively; *F*_ts_ represented the composite tensile force in the HPFRCC side formwork in the tension zone; and *F*_tb_ represented the combined tensile stress in the formwork at the bottom of the HPFRCC in the tension zone, which could be calculated using the following equation:

(8) $$F_{\mathrm{ts}}=2b_{\mathrm{hc}}f_{\mathrm{thc}}\left(h-x_{c}\right)$$

(9) $$F_{\mathrm{tb}}=b_{c}b_{\mathrm{ht}}f_{\mathrm{thc}}$$

where *b*_ht_ and *b*_hc_ were the thicknesses of the bottom shell and side shell of the R/HPFRCC mold shell, respectively; *b*_c_ indicated the width of the lower section of cast-in-place concrete; *x*_c_ represented the height of the compression zone of the section; *f*_thc_ was the cracking stress of HPFRCC material in the tension zone; *F*_cc_ represented the concrete compressive force; and *F*_hc_ represented the compressive force in the HPFRCC in the compression zone, which could be calculated by the integration:

(10) $$F_{\mathrm{cc}}=b_{c}\int_{0}^{x_{c}}\sigma_{\mathrm{cc}}\left(\frac{x\varepsilon_{\mathrm{cu}}}{x_{c}}\right)dx+2b_{\mathrm{hc}}\int_{0}^{h-h_{m}}\sigma_{\mathrm{cc}}\left(\frac{x\varepsilon_{\mathrm{cu}}}{x_{c}}\right)dx$$

(11) $$F_{\mathrm{hc}}=2b_{\mathrm{hc}}\int_{0}^{h_{m}\text{-}c}\sigma_{\mathrm{ch}}\left(\frac{x\varepsilon_{\mathrm{chu}}}{x_{c}}\right)dx$$

where: *σ*_cc_(*ε*) was the concrete compressive stress; *σ*_ch_(*ε*) was the HPFRCC compressive stress, which Equations (4) and (2) can determine; *h*_m_ was the height of the HPFRCC side mold shell; *c* was the distance from the bottom plate of the R/HPFRCC mold shell to the neutralization axis; and *x* was the distance from the position of any point in the pressure zone to the neutralization axis.

Concerning the general reinforced concrete member positive section bending calculation method, the calculation could be simplified according to the principle of equivalence, i.e., the magnitude of the combined force and the point of action was the same and so could be used to replace the theoretical stress graph of the material in the compression zone at the limit state of bearing capacity with the equivalent rectangular stress distribution (Figure 13). In the picture, *α*_c_ was the ratio of the concrete stress in the equivalent compression region to the axial compressive strength value *f*_c_; *α*_h_ was the ratio of the HPFRCC stress value in the equivalent compression zone to the HPFRCC axial compressive strength value *f*_ch_; *β*_c_ was the ratio of the height of the concrete equivalent rectangular stress map to *x*_c_; and *β*_h_ was the ratio of the height of the HPFRCC equivalent force map to *h*_m-c_. When the concrete strength class was not larger than C50, the Concrete Structure Design Code [23] stated that *β*_c_ and *α*_c_ might be considered as zero point eight and one, respectively. *F*_cc_ could then be further deduced from the following expression:

(12) $$F_{\mathrm{cc}}=\alpha_{c}f_{c}\left(b-b_{c}\right)\beta_{c}\left(h-h_{m}\right)+\alpha_{c}f_{c}b_{c}\beta_{c}x_{c}$$

Bringing Equations (8)–(12) into Equation (7), it could be obtained:

(13) $$\begin{array}{l} \alpha_{c}f_{c}\left(b-b_{c}\right)\beta_{c}\left(h-h_{m}\right)+\alpha_{c}f_{c}b_{c}\beta_{c}x_{c}+2Ab_{\mathrm{hc}}f_{\mathrm{ch}}+f_{y}^{\prime }A_{\mathrm{sc}}^{\prime }\\ =2b_{\mathrm{hc}}f_{\mathrm{thc}}\left(h-x_{c}\right)+b_{c}b_{\mathrm{ht}}f_{\mathrm{thc}}+f_{\mathrm{ytz}}A_{\mathrm{stz}}+f_{\mathrm{yt}}A_{\mathrm{st}}+f_{\mathrm{yth}}A_{\mathrm{sth}} \end{array}$$

where: $A=\int_{0}^{h_{m}-c}\frac{\varepsilon_{x}}{\varepsilon_{\mathrm{ch}}}dx$.

The height *x*_c_ of the compression zone of the R/HPFRCC assembled monolithic composite beam section could be solved according to Equation (13):

(14) $$\begin{array}{c} x_{c}=\frac{2b_{\mathrm{hc}}f_{\mathrm{thc}}h+b_{c}b_{\mathrm{ht}}f_{\mathrm{thc}}+f_{\mathrm{ytz}}A_{\mathrm{stz}}+f_{\mathrm{yt}}A_{\mathrm{st}}+f_{\mathrm{yth}}A_{\mathrm{sth}}}{\alpha_{c}f_{c}b_{c}\beta_{c}+2b_{\mathrm{hc}}f_{\mathrm{thc}}}\\ -\frac{\alpha_{c}f_{c}\left(b-b_{c}\right)\beta_{c}\left(h-h_{m}\right)+2Ab_{\mathrm{hc}}f_{\mathrm{ch}}+f_{y}^{\prime }A_{\mathrm{sc}}^{\prime }}{\alpha_{c}f_{c}b_{c}\beta_{c}+2b_{\mathrm{hc}}f_{\mathrm{thc}}} \end{array}$$

The height of the pressurized zone *x*_c_ ≤ 50 mm, calculated according to Equation (14); that was, the first case did not satisfy.

- Neutralization did not shift in R/HPFRCC precast shell (height of pressure zone *x*_c_ ≤ 50 mm)

**Figure 14:**
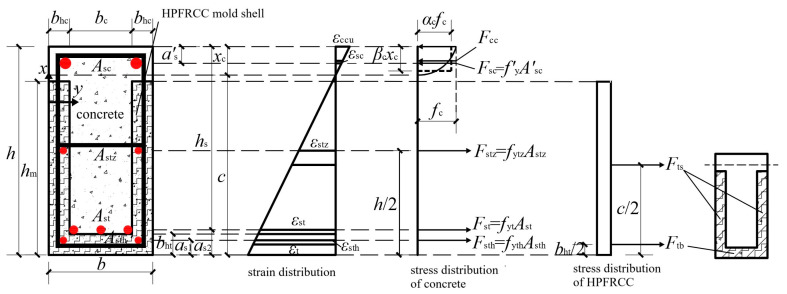
Calculation diagram of bending bearing capacity of R/HPFRCC composite beam under normal section (*x*_c_ ≤ 50 mm).

From the basic assumptions in Section 4.1, for R/HPFRCC assembled monolithic composite beams, considering the role of HPFRCC material, the occurrence of damage in the cross-section occurred on the assumption that the concrete reached the ultimate compressive strain. The cross-section’s stress-strain condition at the moment of damage is depicted in Figure 14, from which the cross-section’s maximum load capacity in bending *M*_u_ may be calculated.

Based on the force equilibrium condition shown in Figure 14, the following expression could be obtained:

(15) $$F_{\mathrm{cc}}+F_{\mathrm{sc}}=F_{\mathrm{ts}}+F_{\mathrm{tb}}+F_{\mathrm{stz}}+F_{\mathrm{st}}+F_{\mathrm{sth}}$$

(16) $$F_{\mathrm{cc}}+f_{y}^{\prime }A_{\mathrm{sc}}^{\prime }=F_{\mathrm{ts}}+F_{\mathrm{tb}}+f_{\mathrm{ytz}}A_{\mathrm{stz}}+f_{\mathrm{yt}}A_{\mathrm{st}}+f_{\mathrm{yth}}A_{\mathrm{sth}}$$

*F*_tb_ represented the HPFRCC tensile force in the tension zone and could be calculated from Equation (9). *F*_ts_ represented the resultant force in the HPFRCC side formwork in the tension zone as follows:

(17) $$F_{\mathrm{ts}}=2b_{\mathrm{hc}}f_{\mathrm{thc}}h_{m}$$

*F*_cc_ represented the concrete compressive force and could be calculated by integration:

(18) $$F_{\mathrm{cc}}=b\int_{0}^{x_{c}}\sigma_{\mathrm{cc}}\left(\frac{x\varepsilon_{\mathrm{cu}}}{x_{c}}\right)dx$$

where *σ*_cc_(*ε*) was the concrete compressive stress, which could be determined by Equation (4), and *x* was the distance from the position of any point in the pressure zone to the neutralization axis.

Concerning the general reinforced concrete member cross-section bending calculation method, the equivalent rectangular stress distribution graph could be used to replace the theoretical stress graph of the material in the compression zone under the load capacity limit state, as shown in Figure 14. Then, *F*_cc_ could be further found from the following expression:

(19) $$F_{\mathrm{cc}}=\alpha_{c}f_{c}b_{c}\beta_{c}x_{c}$$

Substituting Equations (9), (17)–(19) into Equation (16), it could be obtained:

(20) $$\alpha_{c}f_{c}b\beta_{c}x_{c}+f_{y}^{\prime }A_{\mathrm{sc}}^{\prime }=2b_{\mathrm{hc}}f_{\mathrm{thc}}h_{m}+b_{c}b_{\mathrm{ht}}f_{\mathrm{thc}}+f_{\mathrm{ytz}}A_{\mathrm{stz}}+f_{\mathrm{yt}}A_{\mathrm{st}}+f_{\mathrm{yth}}A_{\mathrm{sth}}$$

where *h*_m_ was the height of the HPFRCC mold shell.

The *x*_c_ of the R/HPFRCC assembled monolithic composite beam section could be solved according to Equation (20):

(21) $$x_{c}=\frac{2b_{\mathrm{hc}}f_{\mathrm{thc}}h_{m}+b_{c}b_{\mathrm{ht}}f_{\mathrm{thc}}+f_{\mathrm{ytz}}A_{\mathrm{stz}}+f_{\mathrm{yt}}A_{\mathrm{st}}+f_{\mathrm{yth}}A_{\mathrm{sth}}-f_{y}^{\prime }A_{\mathrm{sc}}^{\prime }}{\alpha_{c}f_{c}b\beta_{c}}$$

Next, moments were taken for the tensile reinforcement ensemble points in the lower mold shell, and the flexural ultimate capacity of the normal section *M*_u_ could be expressed as:

(22) $$\begin{array}{ll} M_{u} & =F_{\mathrm{cc}}\left(h_{0}-\frac{\beta_{c}x_{c}}{2}\right)+f_{y}^{\prime }A_{\mathrm{sc}}^{\prime }\left(h_{0}-a\prime_{s}\right)-f_{\mathrm{ytz}}A_{\mathrm{stz}}\left(h_{0}-\frac{h}{2}\right)-f_{\mathrm{yt}}A_{\mathrm{st}}\left(a_{s2}-a_{s1}\right)\\ & -F_{\mathrm{ts}}\left(\frac{h_{m}}{2}-a_{s1}\right)+F_{\mathrm{tb}}\left(a_{s1}-\frac{b_{\mathrm{ht}}}{2}\right)\\ & =\alpha_{c}f_{c}b\beta_{c}x_{c}\left(h_{0}-\frac{\beta_{c}x_{c}}{2}\right)+f_{y}^{\prime }A_{\mathrm{sc}}^{\prime }\left(h_{0}-a\prime_{s}\right)-f_{\mathrm{ytz}}A_{\mathrm{stz}}\left(h_{0}-\frac{h}{2}\right)\\ & -f_{\mathrm{yt}}A_{\mathrm{st}}\left(a_{s2}-a_{s1}\right)-2b_{\mathrm{hc}}f_{\mathrm{thc}}h_{m}\left(\frac{h_{m}}{2}-a_{s1}\right)+b_{c}b_{\mathrm{ht}}f_{\mathrm{thc}}\left(a_{s1}-\frac{b_{\mathrm{ht}}}{2}\right) \end{array}$$

where *a*_s1_ was the distance from the resultant force point of longitudinal reinforcement in the mold shell to the edge of the tensile zone; *a*_s2_ was the distance from the resultant force point of reinforcement in the cast-in-place concrete to the edge of the tensile zone; *a′*_s_ was the distance from the resultant force point of longitudinal reinforced bars in the compression zone to the compression edge; and *h*_0_ = *h* − *a*_s1_ was the effective height of the section.

## 5. Comparison of Theoretical Calculations and Test Results

Based on the experimental data and the ultimate bearing capacity calculation Formula (22), Table 4 lists the maximum load monitored in the test and the calculated bearing values of the R/HPFRCC precast shell composite beams. The cross-section’s flexural ultimate bearing capacity had a maximum error of 6.52% and a minimum error of 0.99%. The average error of all beams was 3.34%, which might meet our criteria for computation accuracy. The basic assumptions, simplification of the theoretical model, determination of the material parameters, construction quality, material nonlinearities, the accuracy of the test data, and other factors were likely the causes of the deviation between the theoretically calculated values and the test values.

However, since the established theoretical formula ignored the effects of creep and shrinkage, it is only suitable for analyzing bearing capacity under short-term loads. In the future, it is still necessary to study the long-term load, considering the influences of creep and shrinkage, and to modify and improve the calculation formula.

## 6. Conclusions

In this paper, the performances of a new composite beam composed of the reinforced HPFRCC prefabricated shell and post-poured concrete were investigated experimentally and theoretically. The influences of the HPFRCC material used on the prefabricated shells, the reinforcement ratio, the treatment of the post-positioned longitudinal bars, and the different loading modes on the beams’ bending performance were investigated. HPFRCC prefabricated monolithic composite beams’ ultimate flexural capabilities were examined, and a computed flexural capacity model was created. The following were the primary conclusions:

- Compared with the RC precast shell composite beam, the R/HPFRCC prefabricated shell monolithic composite beams presented good bending performance with microcracks and enhanced deformability. The R/HPFRCC composite beams presented excellent crack-developed control ability. The integrity, deformability, and damage resistance of the prefabricated monolithic composite beams could be increased by using HPFRCC material in the prefabricated shells.
- Compared to beam ZSTD1.11R, the peak and yield loads of R/HPFRCC composite beams were 17.2% and 24.6% higher. The bearing capacity of R/HPFRCC beam HPSTD1.21R was 10.3% higher than that of specimen RCSTD1.21R. With an increase of the factor *ρ*_l_, R/HPFRCC composite beam yield and peak loads increased; yield and peak loads increased by 25.6% and 13.5%, respectively, when the longitudinal reinforcement ratio increased by 0.08%.
- Taking the strain-hardening properties of the HPFRCC material into account, the theoretical mechanical study of the bending load capacity of R/HPFRCC prefabricated monolithic composite beams was conducted. A calculating model of the bending capacity of R/HPFRCC composite beams was established. The calculated values of the model and the test values agreed well, with about 3.34% average error.

Due to the limited experimental data, the calculation method proposed in this paper needs to be further verified by experiments. Additionally, only the flexural properties of the composite beam of the structural form were studied. The seismic performance and resilience of the R/HPFRCC precast shell composite members should be investigated in following research.

## Figures and Tables

**Table 1:** Main parameters of composite beam specimens.

Specimen No.	Precast Shell	Load Direction	Shell Material	Longitudinal Reinforcement of Mold Shell	Upper Longitudinal Reinforcement of Beam	Lower Longitudinal Reinforcement of Beam	Longitudinal Rebar Rate/%	Hooped Tendon
HPSTD1.21R	Yes	Bottom in tension	HPFRCC	4C10	2C16	3C12	1.21	A6@70
HPSTD1.13R	Yes	Bottom in tension	HPFRCC	4C10	2C16	2C14	1.13	A6@70
HPSTD1.36R	Yes	Bottom in tension	HPFRCC	4C10	2C16	2C16	1.36	A6@70
HPSTD1.07R	Yes	Bottom in tension	HPFRCC	4C8	2C16	3C12	1.07	A6@70
HPSTD1.21R-W	Yes	Bottom in tension	HPFRCC	4C10	2C16	3C12	1.21	A6@70
HPREV1.46R	Yes	Top in tension	HPFRCC	4C10	3C12	3C16	1.46	A6@70
RCREV1.46R	Yes	Top in tension	RC	4C10	3C12	3C16	1.46	A6@70
RCSTD1.21R	Yes	Bottom in tension	RC	4C10	2C16	3C12	1.21	A6@70
ZSTD1.11R	No	Bottom in tension	-	-	2C16	3C14	1.11	A6@70

Note: In the specimen number, HP represents the mold shell material was HPFRCC, RC represents ordinary reinforced concrete, and Z represents the specimen for the whole pouring form of concrete beams. REV represents reverse loading, and STD represents forward loading. 1.21R represents that the reinforcement ratio of the specimen was 1.21% (other reinforcement ratios were the same).

**Table 2:** Mechanical parameters of reinforced bars.

Type of Rebar	Rebar Diameter (mm)	Yield Strength *f*_y_ (MPa)	Maximum Intensity *f*_u_ (MPa)
HPB300	6	355	506
HRB400	8	410	512
	10	425	626
	12	465	642
	14	442	614
	16	532	698

**Table 3:** The characteristic load value and deflection of the test beam.

No.	Cracking Load /kN	Yield Load /kN	Yield Deflection /mm	55 mm Peak Load at Mid-Span Displacement /kN
HPSTD1.21R	18.0	219.2	11.30	256.6
HPSTD1.13R	18.0	174.5	8.27	226.0
HPSTD1.36R	12.0	233.4	13.14	267.6
HPSTD1.07R	16.0	178.5	10.62	219.4
HPSTD1.21R-W	17.6	208.3	10.56	249.4
HPREV1.46R	18.0	239.8	14.34	295.6
RCREV1.46R	18.0	217.0	9.14	276.4
RCSTD1.21R	28.2	205.6	11.22	232.7
ZSTD1.11R	30.0	176.0	9.95	219.0

**Table 4:** Comparison of theoretical model calculation value and experimental value of the prefabricated monolithic composite beam.

Test Piece	Theoretical Ultimate Bending Moment/kN·m	Measured Ultimate Bending Moment/kN·m	Errors/%
HPSTD1.21R	85.70	89.81	4.58
HPSTD1.13R	80.43	79.10	1.68
HPSTD1.36R	98.39	93.66	4.42
HPSTD1.07R	76.02	76.79	0.99
HPSTD1.21R-W	85.70	87.29	1.82
HPREV1.46R	96.71	103.46	6.52

## Data Availability

The original contributions presented in the study are included in the article. Further inquiries can be directed to the corresponding author.

## References

[1] Ge W.J., Liu C., Zhang Z., Guan Z., Ashour A., Song S., Jiang H., Sun C., Qiu L., Yao S. (2023). Numerical and theoretical research on flexural behavior of steel-precast UHPC composite beams. J. Case Stud. Constr. Mater..

[2] Zhang X., Liu G.H., Shen Z.T., Gao Y.Q., Zhou H.K., Wang Z.Y. (2023). A comprehensive study on the effect of reinforcing methods on the flexural behavior of Concrete-RUHTCC composite beams. J. Eng. Struct..

[3] Li V.C., Stang H., Krenchel H. (1993). Micromechanics of crack bridging in fiber-reinforced concrete. J. Mater. Struct..

[4] Zhang J., Leung C.K.Y., Cheung Y.N. (2006). Flexural performance of layered ECC-concrete composite beam. J. Compos. Sci. Technol..

[5] Hu Z.H., Zhou Y.W., Hu B., Huang X.X., Guo M.H. (2022). Local use of ECC to simultaneously enhance the shear strength and deformability of RC beams. J. Constr. Build. Mater..

[6] Mohammad R., Asadollah R.K., Masoud Z.S. (2022). Experimental Investigation of Deep Beams Containing High-Performance Fiber-Reinforced Cementitious Composite. J. Iran. J. Sci. Technol. Trans. Civ. Eng..

[7] Tian J., Wu X.W., Tan X., Wang W.W., Hu S.W., Du Y.F., Yuan J.Y., Huang W.T., Huang X. (2022). Experimental study and analysis model of flexural synergistic effect of reinforced concrete beams strengthened with ECC. J. Constr. Build. Mater..

[8] Liang X.W., Wang P., Xu M.X., Yu J., Li L. (2019). Flexural behavior and capacity analysis of RC beams with permanent UHPC formwork. J. Eng. Mech..

[9] Zhang P., Xu F., Liu Y., Shamim A.S. (2022). Shear behaviour of composite beams with permanent UHPC formwork and high-strength steel rebar. J. Constr. Build. Mater..

[10] Zhang R., Hu P., Zheng X.H., Cai L.H., Guo R., Wei D.B. (2020). Shear behavior of RC slender beams without stirrups by using precast U-shaped ECC permanent formwork. J. Constr. Build. Mater..

[11] Qin F.J., Wei X.Y., Lu Y.F., Zhang Z.G., Di J., Yin Z.W. (2023). Flexural behaviour of high strength engineered cementitious composites (ECC)-reinforced concrete composite beams. J. Case Stud. Constr. Mater..

[12] Ma R., Guo L.P., Chen Z.K., Li T.Y., Sun W. (2017). Preparation and bending properties of coupled beam made by UHPFRCC and high ductility cementitious composites. J. Southeast Univ. (Nat. Sci. Ed.).

[13] Tosun K., Felekoğlu B., Baradan B. (2012). Multiple cracking response of plasma treated polyethylene fiber reinforced cementitious composites under flexural loading. J. Cem. Concr. Compos..

[14] Tosun-Felekoglu K., Felekoglu B. (2013). Effects of fiber–matrix interaction on multiple cracking performance of polymeric fiber reinforced cementitious composites. J. Compos. Part B Eng..

[15] John K.S., Cascardi A., Verre S., Nadir Y. (2025). RC-columns subjected to lateral cyclic force with different FRCM-strengthening schemes: Experimental and numerical investigation. J. Bull. Earthq. Eng..

[16] Shiotani T., Ogura N., Okude N., Watabe K., Steen C.V., Tsangouri E., Lacidogna G., Czarnecki S., Chai H.K., Yang Y. (2024). Non-destructive inspection technologies for repair assessment in materials and structures. J. Dev. Built Environ..

[17] Mpalaskas C.A., Kytinou K.V., Zapris G.A., Matikas T.E. (2024). Optimizing Building Rehabilitation through Non-destructive Evaluation of Fire-Damaged Steel-Fiber-Reinforced Concrete. J. Sens..

[18] (2019). Standard for Test Methods of Concrete Physical and Mechanical Properties.

[19] Lu T.T., Li Z.L., Pan J.J., Guan K. (2024). Structural Behavior of Precast Monolithic Composite Beams with ECC Prefabricated Shells. J. Build..

[20] Li Y. (2011). Study on Mechanical Performance of High Performance Fiber Reinforced Cement Composite. Ph.D. Thesis.

[21] D’Amato M., Braga F., Gigliotti R., Kunnath S.S., Laterza M. (2012). A numerical general-purpose confinement model for non-linear analysis of R/C members. J. Comput. Struct..

[22] Mander J.B., Priestley M.J.N., Park R. (1988). Theoretical Stress-Strain Model for Confined Concrete. J. Struct. Eng..

[23] (2016). Concrete Structure Design Code.

